# Safety and Immunogenicity of Adenovirus and Poxvirus Vectored Vaccines against a Mycobacterium Avium Complex Subspecies

**DOI:** 10.3390/vaccines9030262

**Published:** 2021-03-16

**Authors:** Pedro M. Folegatti, Amy Flaxman, Daniel Jenkin, Rebecca Makinson, Lucy Kingham-Page, Duncan Bellamy, Fernando Ramos Lopez, Jonathan Sheridan, Ian Poulton, Jeremy Aboagye, Nguyen Tran, Celia Mitton, Rachel Roberts, Alison M. Lawrie, Adrian V. S. Hill, Katie J. Ewer, Sarah Gilbert

**Affiliations:** The Jenner Institute, University of Oxford, Oxford OX3 7DQ, UK; amy.flaxman@ndm.ox.ac.uk (A.F.); daniel.jenkin@ndm.ox.ac.uk (D.J.); rebecca.makinson@ndm.ox.ac.uk (R.M.); lucy.kingham-page@cardiov.ox.ac.uk (L.K.-P.); duncan.bellamy@ndm.ox.ac.uk (D.B.); fernando.ramoslopez@ndm.ox.ac.uk (F.R.L.); jsheridan1@sheffield.ac.uk (J.S.); ian.poulton@ndm.ox.ac.uk (I.P.); jeremy.aboagye@ndm.ox.ac.uk (J.A.); nguyen.tran@ndm.ox.ac.uk (N.T.); mitton@hotmail.co.uk (C.M.); rachel.roberts@ndm.ox.ac.uk (R.R.); alison.lawrie@ndm.ox.ac.uk (A.M.L.); adrian.hill@ndm.ox.ac.uk (A.V.S.H.); katie.ewer@ndm.ox.ac.uk (K.J.E.); sarah.gilbert@ndm.ox.ac.uk (S.G.)

**Keywords:** viral vector, T-Cell, vaccine

## Abstract

Heterologous prime-boost strategies are known to substantially increase immune responses in viral vectored vaccines. Here we report on safety and immunogenicity of the poxvirus Modified Vaccinia Ankara (MVA) vectored vaccine expressing four Mycobacterium avium subspecies paratuberculosis antigens as a single dose or as a booster vaccine following a simian adenovirus (ChAdOx2) prime. We demonstrate that a heterologous prime-boost schedule is well tolerated and induced T-cell immune responses.

## 1. Introduction

Mycobacterium avium subspecies paratuberculosis (MAP) has been associated with and is hypothesized to play a role in auto-immune diseases, such as Crohn’s disease, type-1 diabetes and multiple sclerosis [1].

Heterologous prime-boost strategies are known to substantially increase immune responses in viral vectored vaccines [2]. Adenoviral vectors are known for being exceptional priming agents for cellular and humoral immune responses to vaccine antigens, although anti-vector immunity is also induced which could limit its use as a boosting agent. Poxvirus Modified Vaccinia Ankara (MVA) vectors are highly effective as booster agents to CD4+ and CD8+ T cells. Therefore, immune responses can be optimized through strategies using an adenovirus prime followed by an MVA boost, especially for T-cell targeted vaccines [3,4].

We previously reported on the safety and immunogenicity of the novel recombinant simian adenovirus vector, ChAdOx2, as a vaccine expressing four (MAP) antigens [5]. The 4 MAP genes within the 95 kDa polypeptide fusion construct were selected on the basis of known constitutive secreted expression or predicted presence at the mycobacterial cell surface. Here, we report on safety and immunogenicity of the poxvirus MVA vectored vaccine expressing the same MAP antigens as a single dose or as a booster vaccine following ChAdOx2 prime.

## 2. Materials and Methods

### 2.1. ChAdOx2 HAV and MVA HAV Vaccines

The antigen for both vectored vaccines used in the study consists of a 95kDa fusion construct from four MAP genes which are present in all MAP strains, named HAV: 1589c (AhpC), MAP 1234 (Gsd), 2444c (p12) and 1235 (mpa).

ChAdOx2 has been described elsewhere [6]. In summary, ChAdOx2 consists of a replication-deficient simian adenovirus (E1 and E3 genes deleted) derived from the AdC68 strain.

MVA is a highly attenuated poxvirus vector which has been extensively used as a vaccine on its own since the 1970s [7] and in recent years as a viral vector in vaccine clinical trials for multiple different diseases [8].

ChAdOx2 HAV and MVA HAV were manufactured to current good manufacturing practices by the Clinical Biomanufacturing Facility (University of Oxford, Oxford, UK) and IDT Biologika GmbH (Dessau-Rosslau, Germany), respectively.

### 2.2. Study Design and Participants

**Figure 1:**
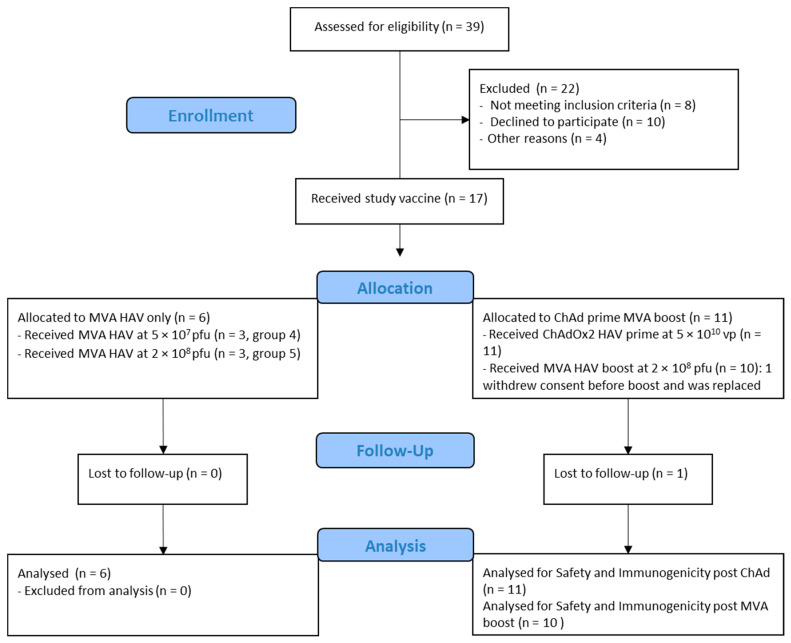
Consolidated Standards of Reporting Trials (CONSORT) diagram.

Eligible volunteers were recruited at the Centre for Clinical Vaccinology and Tropical Medicine, Oxford, UK (Consolidated Standards of Reporting Trials (CONSORT) diagram: Figure 1). Written informed consent was obtained in all cases, and the trial was conducted in accordance with the principles of the Declaration of Helsinki and Good Clinical Practice (GCP). The study was approved in the UK by the regulatory authority and the national research ethics committee. An independent Local Safety Monitor (LSM) provided safety oversight. The trial is registered at www.clinicaltrials.gov (identifier: NCT03027193).

The primary objective was to assess the safety of ChAdOx2 HAV and MVA HAV in healthy adult volunteers administered alone and in a prime-boost regimen by collecting solicited adverse events (AEs) for 7 days, unsolicited and laboratory AEs for 28 days post each vaccine administration and the occurrence of any serious adverse events (SAEs) throughout the trial.

The secondary objective was to assess the immunogenicity of ChAdOx2 HAV and MVA HAV in healthy adult volunteers administered alone and in a prime-boost regimen by ex vivo interferon-gamma (IFN-γ) enzyme-linked immunospot (ELISpot).

### 2.3. Procedures

MVA HAV was administered as a single intramuscular injection into the deltoid at a low dose of 5 × 10^7^ plaque forming units (pfu) in group 4 (*n =* 3), and a standard dose of 2 × 10^8^ pfu in group 5 (*n =* 3). A staggered-enrolment approach was used for the participants in each group and interim safety reviews conducted prior to dose escalation. Group 6 volunteers (*n =* 10) were primed with ChAdOx2 HAV at 5 × 10^10^ viral particles (vp) and received a booster with MVA HAV at 2 × 10^8^ pfu eight weeks later. The dose of ChAdOx2 HAV for the prime-boost group was determined based on safety and immunogenicity responses previously observed.

Blood samples were drawn, and clinical assessments conducted for safety and/or immunology endpoints prior to vaccination at day 0 and subsequently at 2, 7, 14, 28, and 56 days following each vaccine administration. Participants were asked to record any adverse events (AEs) using electronic diaries and were reviewed in clinic during the 28-day follow-up period. Expected and protocol defined local site and systemic reactions were recorded for 7 days. Unsolicited AEs were recorded for 28 days and serious adverse events (SAEs) were recorded throughout the follow-up period.

Severity of AEs was graded using the following criteria: (a) Grade 1 mild (short-lived or mild symptoms with no limitation to usual activity); (b) Grade 2 moderate (mild to moderate limitation in usual activity); and (c) Grade 3 severe (considerable limitation in activity, medication or medical attention required). Unsolicited AEs were reviewed for causality by an independent clinician and events considered possibly, probably or definitively related with the study vaccine were reported. Laboratory AEs were graded using site-specific toxicity tables which were adapted from the US Food and Drug Administration toxicity grading scale.

### 2.4. IFN-γ ELISpot

Responses to vaccination with ChAdOx2 HAV and MVA HAV were assessed by interferon-gamma enzyme-linked immunospot (INF-γ ELISpot) assays using freshly isolated peripheral blood mononuclear cells (PBMC) stimulated with pools of peptides spanning the HAV vaccine construct (Supplementary Table S3). Assays were performed prior to vaccination (day 0), at 14 days, one and two months post each vaccination (days 14, 28 and 56). Methodology was as described previously [5]. Results are expressed as spot forming cells (SFC) per million PBMCs, calculated by subtracting the mean negative control response from the mean of each peptide pool response and then summing the response for the eight peptide pools, or by summing the response for the relevant pools when assessing each of the 4 antigens individually. Each pool contained between 11 and 13 15-mer peptides overlapping by 10 amino acids, spanning the complete vaccine insert. Peptides were pooled so that no pool contained peptides from more than one antigen in the insert. ELISpot plates were excluded if responses were >80 SFC/million PBMC in the negative control (medium only wells) or <800 SFC/million PBMC in the positive control (phytohemagglutinin/staphylococcal enterotoxin B) wells. These quality control (QC) criteria were defined prior to the commencement of sample analysis and 6 samples were excluded from the final dataset, one due to high background in the negative control wells and 5 because of problems with development of the plates.

### 2.5. Statistical Analysis

Safety endpoints are described as frequencies with their respective percentages alongside 95% confidence intervals (CI). Statistical analysis of immunogenicity data was conducted using GraphPad Prism version 9.0 for Mac (GraphPad Software Inc., California, USA). Comparisons of responses between immunogenicity timepoints were made using Wilcoxon matched pairs signed rank test, with *p* < 0.05 considered significant. Correlations were performed using a 2-tailed Spearman’s test.

## 3. Results

### 3.1. Study Population

Between 6 February 2019 and 28 August 2019, 17 healthy adult participants were screened and received either a single dose of MVA HAV (*n =* 6) or a prime dose of ChAdOx2 HAV (*n =* 11) followed by an MVA HAV (*n =* 10) boost 8 weeks apart. Their baseline characteristics are summarized in Table 1. One volunteer received a prime dose of ChAdOx2 HAV but withdrew consent before their booster appointment and was replaced.

### 3.2. Vaccine Safety

ChAdOx2 and MVA were safe and well tolerated in all groups with no serious adverse events reported.

**Figure 2:**
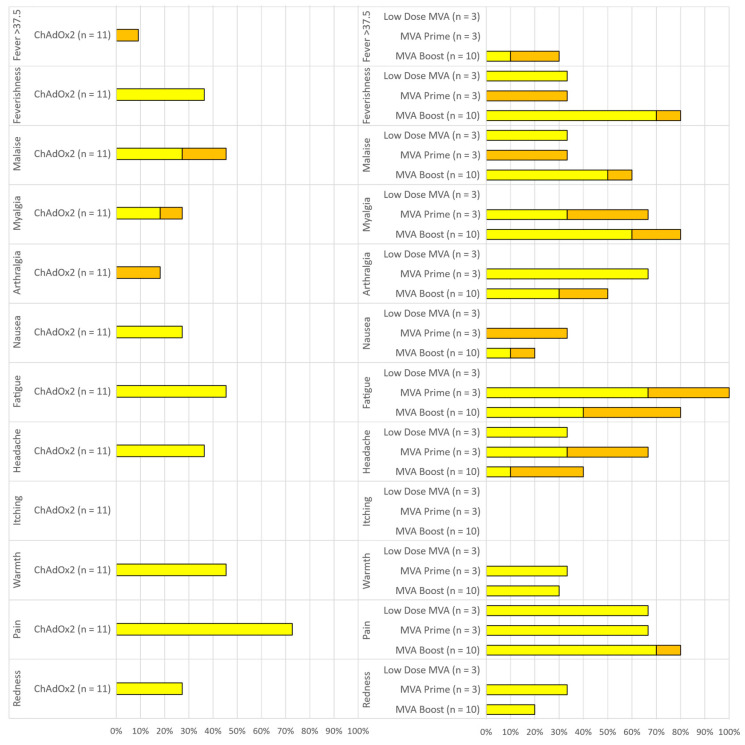
Reactogenicity of ChAdOx2 HAV prime (**left**) and Modified Vaccinia Ankara (MVA) HAV single or booster dose (**right**). Percentage of participants reporting solicited local and systemic adverse events (AEs) within 7 days of vaccination. Yellow *=* mild. Orange = Moderate. Low dose MVA = Group 4 (MVA 2 × 10^7^ pfu prime only), MVA prime = Group 5 (MVA 2 × 10^8^ pfu prime only), MVA Boost = Group 6 (MVA 2 × 10^8^ pfu boost, 56 days after a 5 × 10^10^ vp ChAdOx2 prime).

A total of 121 local and systemic solicited AEs were reported by *n =* 15/17 (88%) participants post either prime or boost. The vast majority of solicited AEs were mild (92/121; 76%, 95%CI 67.7–82.7) or moderate (29/121; 24%, 95%CI 17.2–32.3) and self-limiting in nature. No solicited AEs were graded as severe. All solicited AEs completely resolved within 7 days and 115/121 (95%) of them had their onset within the first 72 h post vaccination (63/121, 52% at D0, 51/121, 42% at D1 and 1/121, <1% at D2). Vaccination arm pain was the most common local AE, reported by *n =* 8/11 (73%) participants after ChAdOx2 HAV and *n =* 12/16 (75%) after MVA and was predominantly mild in severity. The most common systemic solicited AE after ChAdOx2 HAV vaccinations were: malaise *n =* 5/11 (45%) and fatigue *n =* 5/11 (45%) followed by feverishness (4/11, 36%) and headache (4/11, 36%). After any dose of MVA, fatigue (11/16, 69%) was the most common systemic AE followed by feverishness (10/16, 63%) and myalgia (10/16, 63%) then malaise (8/16, 50%). Frequencies of local and systemic solicited AEs reported during the first 7 days are summarized in Figure 2. There were no serious or severe adverse events.

Four participants reported a short-lived temperature above 37.5 °C within the first 48 h post vaccination (1/11, 9% post ChAd in group 6 and 3/10, 33% post MVA in group 6). All febrile episodes resolved within 24 h of onset.

Unsolicited AEs in the 28 days following vaccination considered possibly, probably or definitively related to ChAdOx2 HAV or MVA HAV were predominantly mild in nature and resolved within the follow-up period (Supplementary Table S1). Laboratory AEs considered at least possibly related to the study intervention were self-limiting and predominantly mild in severity (Supplementary Table S2).

### 3.3. Cellular Immunogenicity

**Figure 3:**
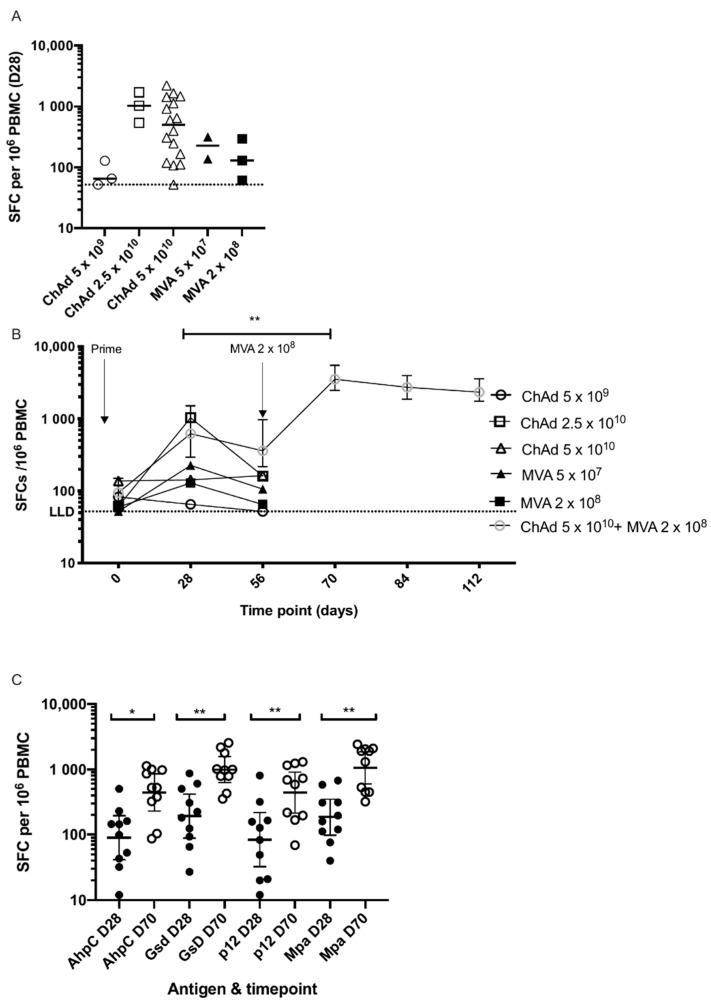
T-Cell immune responses.ELISPOT responses to ChAd and MVA HAV. (**A**) Summed responses to HAV peptide pools at 28 days post prime. Doses shown are vp (viral particles) for ChAdOx2 HAV and pfu (plaque-forming units) for MVA HAV administered to healthy volunteers. Bars represent medians. Dotted line represents ELISPOT lower limit of detection. Lower doses of ChAdOx2 HAV as previously reported also included (5 × 10^9^ and 2.5 × 10^10^ vp). (**B**). Time course of responses to priming vaccination with ChAd or MVA or prime-boost vaccination. Prime only groups were only followed-up for immune responses at 2 months. Medians with inter-quartile ranges are shown. ** *p* < 0.01, 2-tailed Wilcoxon matched pairs test for difference in response between day 28 and day 70 in prime-boost group. (**C**). Responses at day 28 and 70 to individual antigens in the HAV vaccine construct from 10 volunteers in group 6 who were boosted with MVA HAV. Summed responses to peptides are shown with bars representing geometric means with 95% confidence intervals. * *p* < 0.05, ** *p* < 0.01 2-tailed Wilcoxon matched pairs test for difference in response between day 28 and day 70 in the prime-boost group. Doses shown are vp (viral particles) for ChAdOx2 HAV and pfu (plaque-forming units) for MVA HAV.

Prior to vaccination, responses to the HAV antigens were low (median 84.9 SFC) as measured in the INF-γ ELISpot. One month after vaccination with MVA HAV alone, response to vaccination with the lower dose (5 × 10^7^ pfu) and higher dose (2 × 10^8^ pfu) had increased to a median of 226 SFC and 129 SFC, respectively. This compared with medians of 65 SFC, 1033 SFC and 498 SFC in the low (5 × 10^9^ vp), medium (2.5 × 10^10^ vp) and high (5 × 10^10^ vp) doses of ChAdOx2 HAV, respectively (Figure 3A) at the same timepoint. Ten participants that received the higher dose of ChAdOx2 HAV were boosted eight weeks later with the higher dose of MVA HAV. Fourteen days after boosting (D70), responses increased significantly compared with D28 (*p* < 0.01) reaching a median of 3531 SFC and remained high at a median of 2340 SFC at 2 months after boosting (D112, Figure 3B).

Responses to individual antigens in the HAV vaccine construct were assessed in the 10 participants who were primed and boosted. Responses to all four antigens increased significantly after boosting with HAV MVA (Figure 3C). There was no correlation between baseline response (D0) to the HAV construct and the magnitude of the response after priming (D28) or boosting (D70), nor was there a correlation between the response after priming (D28) and boosting (D70).

## 4. Discussion

We have shown that ChAdOx2 HAV and MVA HAV vaccines were safe and well tolerated when given on their own and/or as part of a heterologous prime-boost regimen. The majority of AEs reported were mild or moderate in severity, and all were self-limiting. Mild transient hematological changes from baseline (lymphopenia and neutropenia) were observed, which is in line with what is commonly observed with viral vector vaccines. The profile of adverse events reported here is similar to that for other simian adenovirus and MVA vectored vaccines expressing different antigens [9].

Modest T-cell responses were observed following ChAdOx2 HAV prime, consistent with our previous report, and single dose MVA HAV vaccinations. However, T-cell responses were significantly boosted by MVA HAV following ChAdOx2 HAV prime which persisted above baseline levels for at least two months post boost. Responses were boosted three-fold by administration of MVA HAV and were maintained for at least two months. A heterologous prime-boost approach is, therefore, preferred over prime only strategies with ChAdOx2 and MVA HAV vaccines. T cell, rather than antibody responses are considered to be responsible for protection against intracellular agents, such as those in the Mycobacterium avium complex.

Limitations of this study include the relatively short follow-up period, small sample size and open-labelled, non-randomized, uncontrolled study design. Generalizability of the study findings is limited, as this is a first-in-human study of healthy volunteers.

## 5. Conclusions

In conclusion, ChAdOx2 HAV and MVA HAV were safe and well tolerated with T-cell responses significantly improved and sustained for at least 2 months post boost when given as part of a heterologous prime-boost regimen.

## Figures and Tables

**Table 1:** Summary of baseline characteristics.

Variable	Group 4 (MVA Only Low Dose) (*n* = 3)	Group 5 (MVA Only Standard Dose) (*n* = 3)	Group 6 (Heterologous Prime-Boost) (*n* = 11)	All Groups (*n* = 17)
Age				
Median	22	24	27.5	27
Range	19–23	20–48	21–50	19–50
Sex				
Male—n (%)	0	2 (66.7)	2 (18.2)	4 (23.5)
Female—n (%)	3 (100)	1 (33.3)	9 (81.8)	13 (76.5)
Ethnicity				
White—n (%)	3 (100)	3 (100)	11 (100)	17 (100)
BMI (Kg/m^2^)				
Median	19.7	24	25.7	25.5

Screened participants met the inclusion criteria if aged 18–50. Participants enrolled were aged 19–50.

## Data Availability

The study protocol is available with this publication as part of the supplementary material. Individual participant data may be available upon request directed to the corresponding author, and after approval of a proposal can be shared through a secure online platform.

## Supplementary Materials

The following are available online at https://www.mdpi.com/2076-393X/9/3/262/s1, Table S1. Unsolicited Adverse Reactions (unsolicited adverse events deemed possibly, probably or definitely related to vaccination). Table S2. Laboratory AEs deemed possibly, probably or definitely related to vaccination). Table S3. INF-γ ELISpot peptide pools.
