# Supplementary material for: Safety and Immunogenicity of Adenovirus and Poxvirus Vectored Vaccines against a Mycobacterium Avium Complex Subspecies
Source: Vaccines (Basel). 2021 Mar 16;9(3):262. doi: 10.3390/vaccines9030262 (PMC8000717; doi:10.3390/vaccines9030262)
Supplement: Supplementary file 1 [file vaccines-09-00262-s001.pdf]

Supplementary

Table S1. Unsolicited Adverse Reactions (unsolicited adverse events deemed possibly, probably or definitely related to vaccination).

Description (Meddra PT)	Vaccination	Group	Days since vaccination	Duration of AE (days)	Associated medication	Maximum severity
Joint stiffness	5×10^7 pfu MVA prime	4	1	1	No	mild
Pain in extremity	2×10^8 pfu MVA boost	6	0	2	No	mild
Lymphadenopathy	2×10^8 pfu MVA boost	6	1	3	No	mild
Vaccination site erythema	2×10^8 pfu MVA boost	6	2	1	No	mild
Presyncope	2×10^8 pfu MVA boost	6	0	1	No	moderate
Feeling cold	2×10^8 pfu MVA boost	6	1	1	No	moderate
Myalgia	2×10^8 pfu MVA boost	6	0	2	No	moderate
Dizziness	2×10^8 pfu MVA boost	6	0	1	No	mild
Chills	2×10^8 pfu MVA boost	6	0	1	No	moderate
Dizziness	5×10^{10} vp ChAdOx2 prime	6	0	1	No	mild
Feeling cold	5×10^{10} vp ChAdOx2 prime	6	0	1	No	moderate
Chills	5×10^{10} vp ChAdOx2 prime	6	1	1	No	mild

Table S2. Laboratory AEs deemed possibly, probably or definitely related to vaccination).

Vaccine	AE	timepoint	n participants
ChAdOx2 Prime (n = 11)	Grade 1 Increased alt	D2	1/11
	Grade 1 Reduced haemoglobin	D7	1/11
	Grade 1 Reduced lymphocytes	D2	2/11
	Grade 1 Reduced lymphocytes	D56	1/11
	Grade 1 Reduced neutrophils	D2	3/11
	Grade 1 Reduced white blood count	D2	2/11
MVA Prime (n = 6)	Grade 1 Reduced lymphocytes	D2	3/6
	Grade 1 Reduced neutrophils	D2	1/6
	Grade 1 Reduced neutrophils	D7	1/6
MVA Boost (n = 10)	Grade 1 Reduced lymphocytes	D58 (MVA + 2)	1/10
	Grade 2 Reduced lymphocytes	D58 (MVA + 2)	1/10
	Grade 1 Reduced neutrophils	D58 (MVA + 2)	3/10
	Grade 1 Reduced platelets	D58 (MVA + 2)	1/10
	Grade 1 Reduced white blood count	D58 (MVA + 2)	2/10
	Grade 1 Increased eosinophils	D63 (MVA + 7)	1/10

Table S3. INF-γ ELISpot peptide pools.

Peptide Name	Pool	Peptide Sequence/Description
AhpC1	AHPC Pool 1	LPLLTIGDQFPAYEL
AhpC2		IGDQFPAYELTALIA
AhpC3		PAYELTALIAGDLSK
AhpC4		TALIAGDLSKVDAKQ
AhpC5		GDLSKVDAKQPGDYF
AhpC6		VDAKQPGDYFTTVTS
AhpC7		PGDYFTTVTSEDHAG
AhpC8		TTVTSEDHAGKWRVV
AhpC9		EDHAGKWRVVFFWPK
AhpC10		KWRVVFFWPKDFTGP
AhpC11		FFWPKDFTGPEIATF
AhpC12		DFTGPEIATFGKLND
AhpC13	AHPC Pool 2	EIATFGKLNDEFEDR
AhpC14		GKLNDEFEDRDAQVL
AhpC15		EFEDRDAQVLGVSID
AhpC16		DAQVLGVSIDSEFVH
AhpC17		GVSIDSEFVHFNWRA
AhpC18		SEFVHFNWRAQHEDL
AhpC19		FNWRAQHEDLKNLPF
AhpC20		QHEDLKNLPFPMLSD
AhpC21		KNLPFPMLSDIKREL
AhpC22		PMLSDIKRELSLATG
AhpC23		IKRELSLATGVLNAD
AhpC24		SLATGVLNADGVADR
AhpC25	AHPC Pool 3	VLNADGVADRATFIV
AhpC26		GVADRATFIVDPNNE
AhpC27		ATFIVDPNNEIQFVS
AhpC28		DPNNEIQFVSVTAGS
AhpC29		IQFVSVTAGSVGRNV
AhpC30		VTAGSVGRNVEEVLR
AhpC31		VGRNVEEVLRVLDAL
AhpC32		EEVLRVLDALQSDEL
AhpC33		VLDALQSDELCACNW
AhpC34		QSDELCACNWRKGDP
AhpC35		CACNWRKGDPTLNAT
AhpC36		RKGDPTLNATELLKA
AhpC37		TLNATELLKASA
Gsd1	GSD Pool 1	LGSIVGQTYREVEVV
Gsd2		GQTYREVEVVLVDGG
Gsd3		EVEVVLVDGGSTDRT
Gsd4		LVDGGSTDRTLDIAN
Gsd5		STDRTLDIANSFRPE
Gsd6		LDIANSFRPELGSRL
Gsd7		SFRPELGSRLVVHSG
Gsd8		LGSRLVVHSGPDDGP
Gsd9		VVHSGPDDGPYDAMN
Gsd10		PDDGPYDAMNRGVGV
Gsd11		YDAMNRGVGVATGEW
Gsd12		RGVGVATGEWVLFLG
Gsd13	GSD Pool 2	ATGEWVLFLGADDTL
Gsd14		VLFLGADDTLYEPTT
Gsd15		ADDTLYEPTTLAQVA
Gsd16		YEPTTLAQVAAFLGD
Gsd17		LAQVAAFLGDHAASH

Gsd18		AFLGDHAASHLVYGD
Gsd19		HAASHLVYGDVVMRS
Gsd20		LVYGDVVMRSTKSRH
Gsd21		VVMRSTKSRHAGPFD
Gsd22		TKSRHAGPFDLDRLL
Gsd23		AGPFDLDRLLFETNL
Gsd24		LDRLLFETNLCHQSI
Gsd25		FETNLCHQSIFYRRE
Gsd26		CHQSIFYRRELFDGI
Gsd27		FYRRELFDGIGPYNL
Gsd28		LFDGIGPYNLRYRVW
Gsd29		GPYNLRYRVWADWDF
Gsd30	GSD Pool 3	RYRVWADWDFNIRCF
Gsd31		ADWDFNIRCFSNPAL
Gsd32		NIRCFSNPALITRYM
Gsd33		SNPALITRYMDVVIS
Gsd34		ITRYMDVVISEYNDM
Gsd35		DVVISEYNDMTGFSM
Gsd36		EYNDMTGFSMRQGTD
Gsd37		TGFSMRQGTDKEFRK
Gsd38		RQGTDKEFRKRLPMY
Gsd39		KEFRKRLPMYFWVAG
Gsd40		RLPMYFWVAGWETCR
Gsd41		FWVAGWETCRRMLAF
Gsd42	GSD Pool 4	WETCRRMLAFLKDKE
Gsd43		RMLAFLKDKENRRLA
Gsd44		LKDKENRRLALRTRL
Gsd45		NRRLALRTRLIRVKA
Gsd46		LRTRLIRVKAVSKER
Gsd47		IRVKAVSKERSAEP
p12-1		RIRRHRHAEIILSMP
p12-2		RHAEIILSMPGFGVI
p12-3		ILSMPGFGVILGAEF
p12-4		GFGVILGAEFLAATG
p12-5		LGAEFLAATGGDMAA
p12-6		LAATGGDMAAFASAD
p12-7	P12 Pool 1	GDMAAFASADRLAGV
p12-8		FASADRLAGVAGLAP
p12-9		RLAGVAGLAPVPRDS
p12-10		AGLAPVPRDSGRISG
p12-11		VPRDSGRISGNLKRP
p12-12		GRISGNLKRPRRYDR
p12-13		NLKRPRRYDRRLLRA
p12-14		RRYDRRLLRACYLSA
p12-15		RLLRACYLSALVSIR
p12-16		CYLSALVSIRTDPSS
p12-17		LVSIRTDPSSRTYYD
p12-18		TDPSSRTYYDRKRTE
p12-19		RTYYDRKRTEGKRHT
p12-20	P12 Pool 2	RKRTEGKRHTQAVLA
p12-21		GKRHTQAVLALARRR
p12-22		QAVLALARRRLNVLW
p12-23		LARRRLNVLWAMLRD
p12-24		LNVLWAMLRDHAVYH
p12-25		AMLRDHAVYHPATTT

p12-26		HAVYHPATTTAAA
mpa1	MPA Pool 1	RLKLRRGERPMSLGQ
mpa2		RGERPMSLGQVFDPR
mpa3		MSLGQVFDPRANALH
mpa4		VFDPRANALHSFPLT
mpa5		ANALHSFPLTGRMPW
mpa6		SFPLTGRMPWAPFIV
mpa7		GRMPWAPFIVSSWLR
mpa8		APFIVSSWLRNPHPA
mpa9		SSWLRNPHPAQYFTA
mpa10		NPHPAQYFTARCLRI
mpa11		QYFTARCLRILPGLW
mpa12		RCLRILPGLWIGAQG
mpa13	MPA Pool 2	LPGLWIGAQGGSAAK
mpa14		IGAQGGSAAKLLMSG
mpa15		GSAAKLLMSGAPIEY
mpa16		LLMSGAPIEYVLKDS
mpa17		APIEYVLKDSAVWMF
mpa18		VLKDSAVWMFKFDIG
mpa19		AVWMFKFDIGGTPRD
mpa20		KFDIGGTPRDIPVAG
mpa21		GTPRDIPVAGIWNGS
mpa22		IPVAGIWNGSLWTPA
mpa23		IWNGSLWTPAWGGIH
mpa24		LWTPAWGGIHAIASN
mpa25	MPA Pool 3	WGGIHAIASNAYQFR
mpa26		AIASNAYQFRNVIPA
mpa27		AYQFRNVIPARWSVS
mpa28		NVIPARWSVSSAVLP
mpa29		RWSVSSAVLPNYRLV
mpa30		SAVLPNYRLVAALPM
mpa31		NYRLVAALPMAYHNQ
mpa32		AALPMAYHNQRMRFR
mpa33		AYHNQRMRFRTDLSY
mpa34		RMRFRTDLSYGVYGF
mpa35		TDLSYGVYGFAEINP
mpa36		GVYGFAEINPIALVE
mpa37		AEINPIALVEKPALS
mpa38	MPA Pool 4	IALVEKPALSWKSRL
mpa39		KPALSWKSRLRRKNS
mpa40		WKSRLRRKNSSIALA
mpa41		RRKNSSIALANMEDG
mpa42		SIALANMEDGGSVGR
mpa43		NMEDGGSVGRSNDIP
mpa44		GSVGRSNDIPGRRAR
mpa45		SNDIPGRRARFIGEK
mpa46		GRRARFIGEKAEDPP
mpa47		FIGEKAEDPPAPSPR
mpa48		AEDPPAPSPRPALRI
mpa49		APSPRPALRIPNPLL
mpa50		PALRIPNPLLGLD
